# “Monocept”: A Brief Report of Congenital Absence of the Long Head of the Biceps Tendon and Literature Review

**DOI:** 10.1155/2017/1090245

**Published:** 2017-07-02

**Authors:** Benjamin A. Winston, Katlyn Robinson, Dennis Crawford

**Affiliations:** Department of Orthopaedics and Rehabilitation, Oregon Health & Science University, Portland, OR, USA

## Abstract

The long head of the biceps tendon plays an important role in shoulder stability and its functional absence has been shown to contribute to glenohumeral instability. Congenital absence of the long head to the biceps tendon is rare, although described in the literature. We report the case of an 18-year-old recreational athlete with recurrent shoulder instability and congenital absence of the long head of the biceps tendon (which we term “monocept”) and mild ipsilateral upper extremity hemimelia. The patient was treated surgically with posterior capsular shift with anterior Bankart repair without complication. At 16-month follow-up the patient has returned to recreational activity and has had an 11.37-point improvement in his DASH score. The authors suggest that patients with this uncommon anatomic anomaly and clinical shoulder instability are more likely to require surgical treatment.

## 1. Introduction

Congenital absence of the long head of the biceps (LHB) tendon is an uncommon anatomic anomaly [1, 2]. The LHB plays an important functional role in glenohumeral stability, acting dynamically through the range of motion as well as via depression of the humeral head and serving as anchorage point of the superior glenoid labrum [3]. When damaged and functionally compromised (e.g., superior labral tears), shoulder instability is frequently described. We report a case of a patient with a congenital absence of the LHB tendon presenting with recurrent shoulder instability and the presence of only the short head of the biceps tendon. We coin the term “monocept,” to describe this congenital anomaly.

## 2. Case Presentation

**Figure 1:**
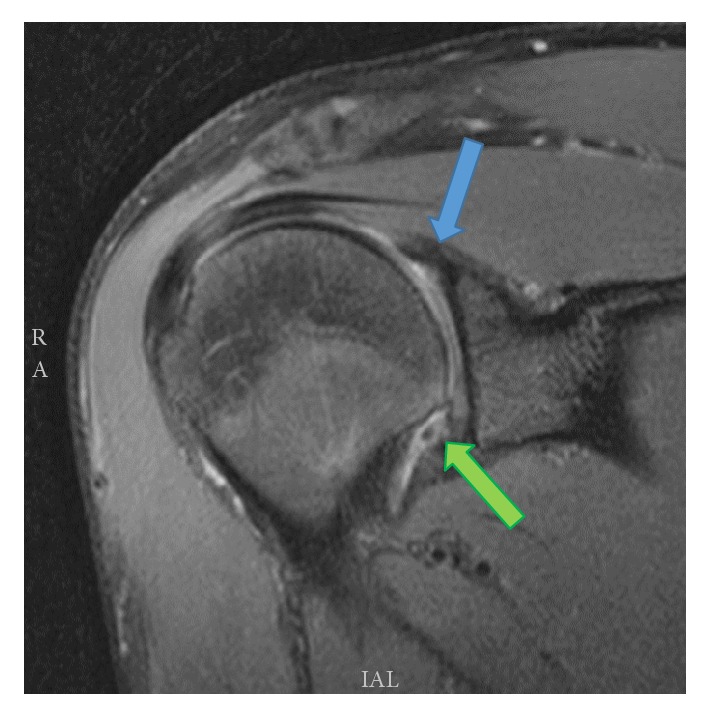
Coronal T2 weighted MRI demonstrating absence of the long head of the biceps tendon (blue arrow) and inferior labral tear (green arrow).

**Figure 2:**
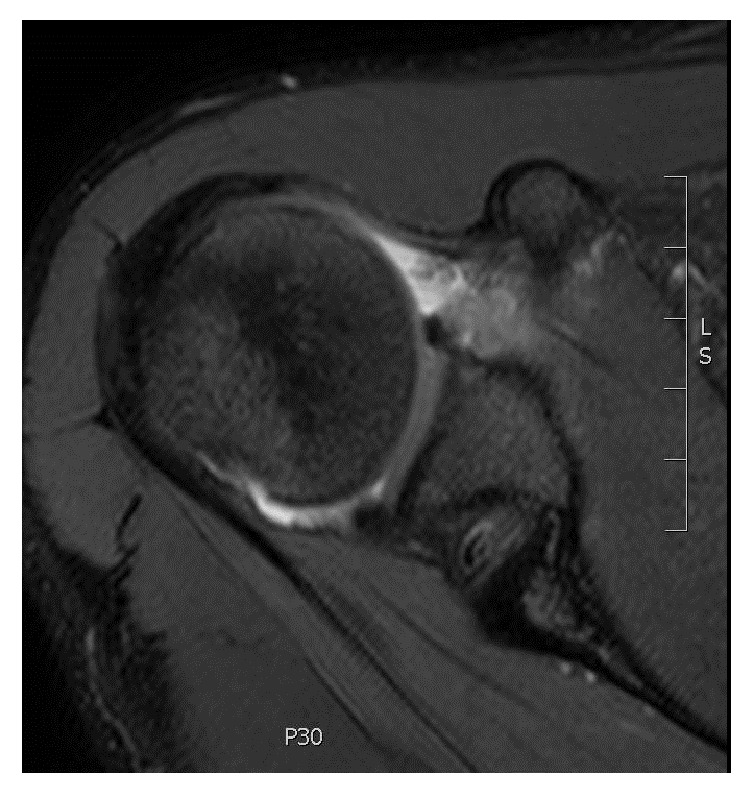
Axial T2 weighted MRI demonstrating absence of the bicipital groove.

This 18-year-old, right-hand dominant male college freshman was referred to our clinic for evaluation of recurrent right shoulder dislocations. He had no history of connective tissue disorder and was otherwise healthy. His first dislocation event occurred 2 years prior while rock climbing and simply reaching forward and laterally to his right. No significant causative trauma was reported in association. He sought no medical treatment as the shoulder was reduced on sight. The patient reported that the dislocations were becoming more frequent and the most recent event occurred while practicing Jiu Jitsu, again while simply reaching without significant forces. Dislocations continued to occur despite rehabilitation exercises. On physical exam, he was noted to have no significant muscle atrophy or scapular dyskinesis. Notable on examination, he was found to have anterior apprehension, a positive inferior sulcus sign (compared to the uninvolved limb), and a mild posterior instability to load shift testing. On observation, the patient also showed signs of a minimal right upper extremity hemimelia despite the fact that this was his dominant arm and that he was a recreational athlete. Most apparent, his right hand was minimally, although noticeably, smaller than left, a finding not previously reported in unilateral congenital absence of the LHB tendon. His preoperative DASH score was 29.55. Magnetic Resonance Imaging (MRI) without contrast of the affected shoulder indicated an anterior Bankart type labral lesion, small nonengaging Hill-Sachs lesion, and most atypically nonvisualization of the biceps tendon (Figure 1) and absence of the bicipital groove (Figure 2).

**Figure 3:**
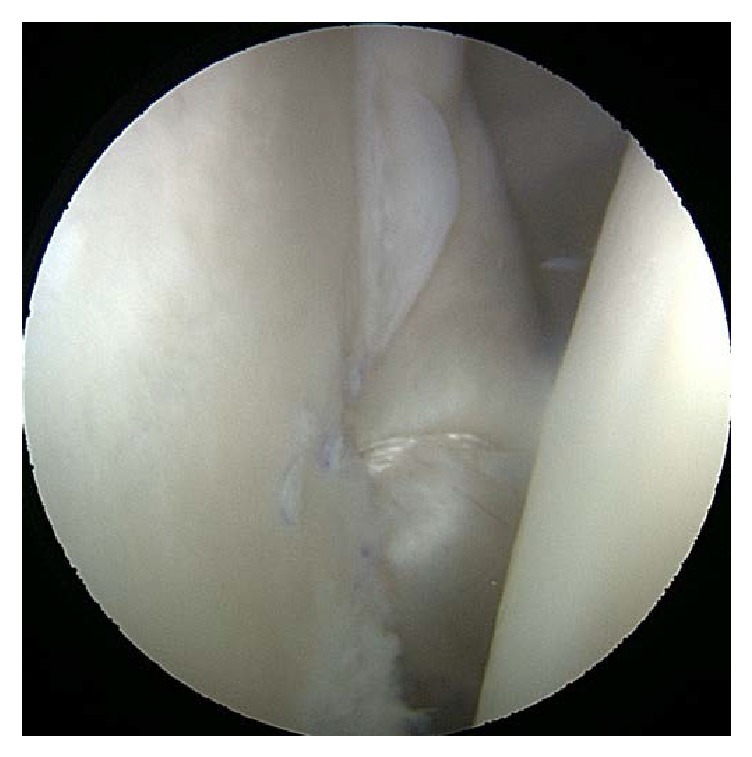
Final intraoperative arthroscopic image after capsular shift.

**Figure 4:**
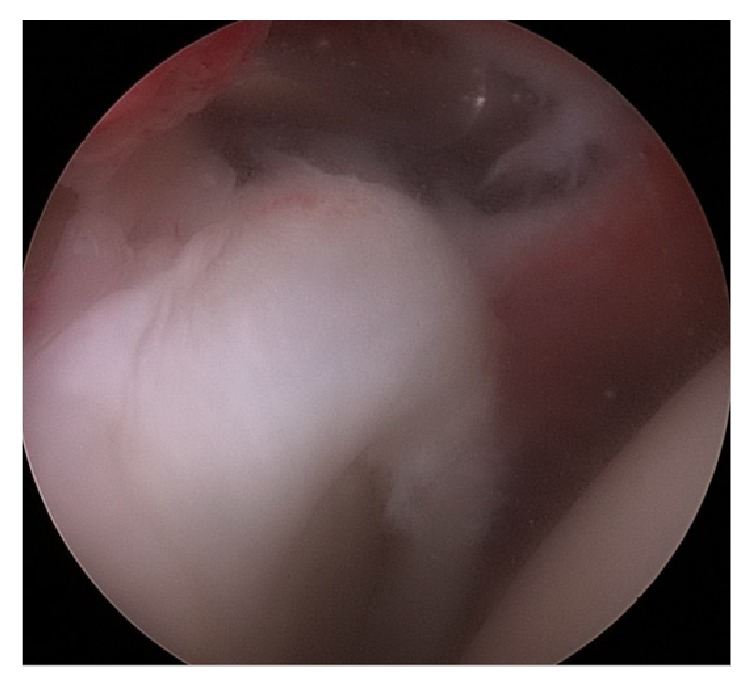
Intraoperative arthroscopic image from the posterior portal, demonstrating absence of origin of the long head of the biceps tendon and presence of the superior labrum.

Arthroscopic shoulder stabilization was recommended and an anterior and posterior capsular shift with anterior Bankart repair were performed (Figure 3). The posterior capsular shift was added to the stabilization procedure due to assessment of subtle multidirectional type instability. The rotator interval was not closed as this was felt to be unnecessary. During the procedure, arthroscopic evaluation confirmed the absence of the long head of the biceps tendon and complete apparently normal superior labrum (Figure 4). His DASH score at his 2-week postoperative follow-up appointment was 20.45. The patient had an uneventful recovery and at 3-month follow-up has returned to routine daily activity (including recreational sporting) without instability symptoms or dislocation events. At latest follow-up (16 months postoperatively) his DASH score was 18.18.

## 3. Discussion

There are several common variants of the origin of the LHB tendon which are typically benign. In contrast, congenital absence of the LHB tendon has a higher reported association with shoulder instability as well as with other congenital abnormalities including VATER syndrome, spina bifida, and congenital limb abnormalities [2, 4]. The association with other congenital anomalies is felt to be the result of a fetal insult at the sixth or seventh week of gestation during biceps differentiation [2, 5]. Although less common, congenital absence of the LHB tendon may occur without any associated congenital anomalies or shoulder instability [2, 6].

When the absence of the LHB tendon is congenital, the bicipital groove is absent or hypoplastic, and therefore, the presence of a bicipital groove visualized on MRI is more consistent with a rupture [3]. It should be noted that a shallow bicipital groove may also result from osteophytes in association with rotator cuff tear. However, in this case the groove tends to be irregular rather than smooth and shallow as is seen in congenital absence [7]. As well, absence of the biceps in the groove can occur for a number of reasons and is not an uncommon finding on MRI. In this case, MRI did indeed demonstrate the absence of the bicipital groove, indicating a congenital absence. Familiarity of the association of a “monocept” type anatomy with shoulder instability in subtle cases of upper extremity dysmorphism may help explain low energy dislocation and provide for a prompt recognition of etiology and treatment planning. Glueck et al. described a similar case in which the congenital absence of the LHB tendon was identified intraoperatively in a 25-year-old patient with multidirectional instability [1]. They report successful treatment with thermal capsulorrhaphy at 1.5-year follow-up. While there is a paucity of literature to guide treatment, both thermal capsulorrhaphy as described by Glueck et al. and capsular shift as in this case report appear to be reasonable treatment options. The authors suggest that patients with this anomaly and clinical shoulder instability are more likely to require surgical treatment.
